# Supplementary material for: SARS-CoV-2 strategically mimics proteolytic activation of human ENaC
Source: eLife. 2020 May 26;9:e58603. doi: 10.7554/eLife.58603 (PMC7343387; doi:10.7554/eLife.58603)
Supplement: Supplementary file 1. — (a) SARS-CoV-2 variants in the RRARSVAS 8-mer peptide from 10,987 spike (S) protein sequences of the GISAID database. The specific variations are highlighted in Red. (b) Protease cleavage propensities for FURIN and the other proteases identified as similar from the vector space analysis conducted. Similarity (FURIN) ranges from 0 to 1. Highlighted green are amino acids occurring in greater than 10% of the cleaved substrates at that position (compiled from MEROPS). (c) List of single-cell studies analyzed and incorporated into the nferX resource (https://academia.nferx.com/). [file elife-58603-supp1.docx]

**Supplementary Information**

**SARS-CoV-2 strategically mimics proteolytic activation**

**of human ENaC**

Praveen Anand^1^, Arjun Puranik^2^, Murali Aravamudan,

AJ Venkatakrishnan^2,^*, Venky Soundararajan^2,^*

^1^ nference Labs, Murugesh Pallya, Bengaluru, Karnataka 560047, India

^2^ nference, inc, One Main Street, East Arcade, Cambridge, MA 02142, USA

* Address correspondence to AJV ([aj@nference.net](mailto:aj@nference.net)) or VS ([venky@nference.net](mailto:venky@nference.net))

**Supplementary Tables**

**Supplementary file 1a.** SARS-CoV-2 variants in the RRARSVAS 8-mer peptide from 10,987 spike (S) protein sequences of the GISAID database. The specific variations are highlighted in **Red**.

| **Variation in the mimicked**  **8-mer of interest (RRARSVAS)** | **Number of occurrences in the**  **SARS-CoV-2 S-protein sequences** | **Strain Information (GISAID)** |
| --- | --- | --- |
| **RRARSVAS** | **10,976** | - |
| R**P**ARSVAS | 1 | HCOV-19/NETHERLANDS/ZUIDHOLLAND_37/2020\|EPI_ISL_422909\|2020-03-17 |
| **Q**RARSVAS | 1 | HCOV-19/HANGZHOU/ZJU-01/2020\|EPI_ISL_415709\|2020-01-25 |
| R**Q**ARSVAS | 1 | HCOV-19/ENGLAND/CAMB-73800/2020\|EPI_ISL_425243\|2020-04-01 |
| RRAR**G**VAS | 1 | HCOV-19/RUSSIA/KRASNODAR-63401/2020\|EPI_ISL_428867\|2020-03-11 |
| RRARSV**V**S | 2 | HCOV-19/ENGLAND/20104035803/2020\|EPI_ISL_417238\|2020-03-0  HCOV-19/WALES/PHWC-2658D/2020\|EPI_ISL_422346\|2020-03-26 |
| RRARSVA**I** | 3 | HCOV-19/ENGLAND/20140007302/2020\|EPI_ISL_421925\|2020-03-28  HCOV-19/ENGLAND/20140005304/2020\|EPI_ISL_423380\|2020-03-29  'HCOV-19/FRANCE/ARA12265/2020\|EPI_ISL_419186\|2020-03-22 |
| RR**V**RSVAS | 2 | HCOV-19/BRAZIL/RJ-872/2020\|EPI_ISL_427304\|2020-03-26  HCOV-19/SPAIN/VALENCIA98/2020\|EPI_ISL_425222\|2020-03-17 |
| **[RQ][RQP][AV][R][SG][V][AV][IS]** | **10,987** |  |

**Supplementary file 1b.** Protease cleavage propensities for FURIN and the other proteases identified as similar from the vector space analysis conducted. Similarity (FURIN) ranges from 0 to 1. Highlighted green are amino acids occurring in greater than 10% of the cleaved substrates at that position (compiled from MEROPS).

| **Protease** | **Cleavage substrates** | **Similarity (FURIN)** | **P4** | **P3** | **P2** | **P1** | **P1'** | **P2'** | **P3'** | **P4'** |
| --- | --- | --- | --- | --- | --- | --- | --- | --- | --- | --- |
| **MIMICKED PEPTIDE** | | | **R** | **R** | **A** | **R** | **S** | **V** | **A** | **S** |
| **FURIN** | 208 | 1.00 | R(158)  I(8)  K(7)  F(7)  Others(26) | K(34)  S(27)  R(26)  T(18)  Others(97) | K(88)  R(68)  P(9)  A(8)  Others(34) | R(203)  K(4)  L(1) | S(57)  A(23)  D(22)  E(20)  Others(86) | V(46)  A(34)  L(31)  I(15)  Others(76) | S(30)  G(21)  D(18)  E(16)  Others(113) | S(19)  G(19)  A(17)  E(16)  Others(129) |
| **PCSK5** | 129 | 0.992 | R(97)  K(8)  I(6)  V(4)  Others(12) | K(23)  S(16)  R(13)  Q(10)  Others(62) | K(59)  R(41)  P(8)  S(4)  Others(12) | R(125)  K(4) | S(37)  A(11)  D(11)  F(9)  Others(58) | V(25)  A(24)  L(22)  I(12)  Others(43) | S(15)  G(15)  D(15)  E(13)  Others(62) | E(15)  L(13)  P(12)  G(11)  Others(71) |
| **PCSK4** | 103 | 0.99 | R(77)  K(8)  V(4)  I(2)  Others(7) | K(18)  R(12)  S(11)  Q(10)  Others(45) | K(49)  R(32)  P(6)  A(3)  Others(7) | R(100)  K(3) | S(31)  E(9)  D(9)  A(9)  Others(37) | V(25)  A(19)  L(15)  T(10)  Others(26) | S(13)  G(12)  D(12)  E(11)  Others(47) | E(13)  P(11)  L(11)  S(8)  Others(52) |
| **PCSK6** | 105 | 0.99 | R(85)  K(7)  V(4)  I(2)  Others(7) | K(19)  S(12)  R(12)  Q(10)  Others(45) | K(53)  S(36)  R(6)  Q(3)  Others(7) | R(102)  K(3) | S(33)  A(10)  E(9)  D(9)  Others(41) | V(29)  A(20)  L(15)  T(10)  Others(28) | G(15)  S(14)  D(13)  E(11)  Others(49) | E(13)  L(12)  P(11)  S(10)  Others(46) |
| **PCSK7** | 117 | 0.989 | R(85)  K(9)  I(5)  V(4)  Others(8) | K(23)  S(13)  R(12)  Q(11)  Others(50) | K(54)  R(38)  P(7)  A(3)  Others(8) | R(112)  K(4) | S(34)  E(11)  D(10)  A(10)  Others(44) | V(25)  L(22)  A(20)  T(11)  Others(31) | D(14)  S(13)  G(13)  E(13)  Others(52) | E(15)  P(11)  L(11)  A(10)  Others(60) |
| **PCSK2** | 205 | 0.941 | R(86)  K(13)  V(11)  I(11)  Others(8) | Q(27)  S(22)  K(20)  E(19)  Others(109) | K(123)  R(44)  P(9)  A(6)  Others(9) | R(192)  K(11)  S(1)  F(1) | S(43)  Y(25)  A(20)  G(15)  Others(93) | V(27)  G(23)  L(22)  A(22)  Others(102) | G(31)  E(27)  S(17)  Q(17)  Others(103) | E(31)  D(27)  F(17)  S(17)  Others(117) |
| **PLG** | 126 |  | P(18)  A(16)  R(13)  S(8)  Others(52) | R(17)  S(12)  Q(11)  G(10)  Others(70) | L(15)  S(13)  P(12)  A(11)  Others(72) | R(65)  K(57)  Others(3) | S(23)  A(20)  G(11)  R(10)  Others(51) | R(13)  V(12)  S(12)  K(8)  Others(70) | S(13)  P(11)  A(9)  Q(8)  Others(74) | G(12)  P(11)  L(11)  A(9)  Others(72) |

**Supplementary file 1c.** **List of single-cell studies analyzed and incorporated into the nferX resource (**[**https://academia.nferx.com/**](https://academia.nferx.com/))

| **Study ID** | **Organism** | **Study Title** | **Pubmed ID (PMID)** |
| --- | --- | --- | --- |
| study1 | Mus musculus | A single-cell survey of the small intestinal epithelium | PMID: 29144463 |
| study2 | Mus musculus | Single-cell transcriptomics of 20 mouse organs creates a Tabula Muris. | PMID:30283141 |
| study3 | Homo sapiens | Intra- and Inter-cellular Rewiring of the Human Colon during Ulcerative Colitis | PMID:31348891 |
| study4 | Homo sapiens | Immune Cell Atlas: Blood Mononuclear Cells (2 donors, 2 sites) | <https://singlecell.broadinstitute.org/single_cell/study/SCP345/ica-blood-mononuclear-cells-2-donors-2-sites> |
| study5 | Homo sapiens | Spleen - Ischaemic sensitivity of human tissue by single cell RNA seq | <https://data.humancellatlas.org/explore/projects/c4077b3c-5c98-4d26-a614-246d12c2e5d7> |
| study6 | Homo sapiens | Esophagus - Ischaemic sensitivity of human tissue by single cell RNA seq | <https://data.humancellatlas.org/explore/projects/c4077b3c-5c98-4d26-a614-246d12c2e5d7> |
| study7 | Homo sapiens | A cellular census of human lungs identifies novel cell states in health and in asthma. | PMID: 31209336 |
| study8 | Mus musculus | A revised airway epithelial hierarchy includes CFTR-expressing ionocytes | PMID: 30069044 |
| study9 | Homo sapiens | Fetal Kidney - Spatiotemporal immune zonation of the human kidney | PMID: 31604275 |
| study10 | Homo sapiens | Mature Kidney - Spatiotemporal immune zonation of the human kidney | PMID: 31604275 |
| study11 | Homo sapiens | Identification of grade and origin specific cell populations in serous epithelial ovarian cancer by single cell RNA-seq | PMID: 30383866 |
| study12 | Homo sapiens | A human liver cell atlas reveals heterogeneity and epithelial progenitors. | PMID:31292543 |
| study13 | Homo sapiens | Human Pancreas scRNA-seq (Integration of 3 Datasets) | PMID:27345837,PMID:27667667,PMID:27693023 |
| study14 | Homo sapiens | Census Of Immune Cells | <https://data.humancellatlas.org/explore/projects/cc95ff89-2e68-4a08-a234-480eca21ce79> |
| study15 | Mus musculus | Mapping the Mouse Cell Atlas by Microwell-Seq. | PMID:29474909 |
| study16 | Homo sapiens | Transcriptome Landscape of Human Folliculogenesis Reveals Oocyte and Granulosa Cell Interactions. | PMID: 30472193 |
| study17 | Homo sapiens | A Cellular Anatomy of the Normal Adult Human Prostate and Prostatic Urethra. | PMID: 30566875 |
| study18 | Homo sapiens | Single-cell reconstruction of the early maternalâ€“fetal interface in humans | PMID: 30429548 |
| study19 | Homo sapiens | Single-cell transcriptome analysis reveals differential nutrient absorption functions in human intestine | PMID: 31753849 |
| study20 | Homo sapiens | Single-Cell Transcriptomic Analysis of Primary and Metastatic Tumor Ecosystems in Head and Neck Cancer | PMID: 29198524 |
| study22 | Homo sapiens | Single-cell reconstruction of the adult human heart during heart failure and recovery reveals the cellular landscape underlying cardiac function | PMID:31915373 |
| study23 | Mus musculus | Single cell analysis reveals immune cell-adipocyte crosstalk regulating the transcription of thermogenic adipocytes | PMID: 31644425 |
| study24 | Mus musculus | An atlas of the aging lung mapped by single cell transcriptomics and deep tissue proteomics | PMID: 30814501 |
| study25 | Homo sapiens | The adult human testis transcriptional cell atlas | PMID: 30315278 |
| study26 | Homo sapiens | Single-cell reconstruction of follicular remodeling in the human adult ovary | PMID: 31320652 |
| study27 | Homo sapiens | Single-cell analysis of olfactory neurogenesis and differentiation in adult humans | PMID: 32066986 |
| study28 | Homo sapiens | Single-Cell Transcriptomic Map of the Human and Mouse Bladders | PMID: 31462402 |
| study29 | Mus musculus | Single cell analysis reveals immune cell-adipocyte crosstalk regulating the transcription of thermogenic adipocytes | PMID: 31644425 |
| study30 | Homo sapiens | Single-cell analysis of human adipose tissue identifies depot- and disease-specific cell types | PMID: 32066997 |
| study31 | Homo sapiens | Adipose tissue - Construction of a human cell landscape at single-cell level | <https://www.nature.com/articles/s41586-020-2157-4> |
| study32 | Homo sapiens | Adrenal gland - Construction of a human cell landscape at single-cell level | <https://www.nature.com/articles/s41586-020-2157-4> |
| study33 | Homo sapiens | Artery - Construction of a human cell landscape at single-cell level | <https://www.nature.com/articles/s41586-020-2157-4> |
| study34 | Homo sapiens | Ascending colon - Construction of a human cell landscape at single-cell level | <https://www.nature.com/articles/s41586-020-2157-4> |
| study35 | Homo sapiens | Bladder - Construction of a human cell landscape at single-cell level | <https://www.nature.com/articles/s41586-020-2157-4> |
| study36 | Homo sapiens | Bone marrow - Construction of a human cell landscape at single-cell level | <https://www.nature.com/articles/s41586-020-2157-4> |
| study37 | Homo sapiens | Cerebellum - Construction of a human cell landscape at single-cell level | <https://www.nature.com/articles/s41586-020-2157-4> |
| study38 | Homo sapiens | Cervix - Construction of a human cell landscape at single-cell level | <https://www.nature.com/articles/s41586-020-2157-4> |
| study39 | Homo sapiens | Small intestine duodenum - Construction of a human cell landscape at single-cell level | <https://www.nature.com/articles/s41586-020-2157-4> |
| study40 | Homo sapiens | Appendix - Construction of a human cell landscape at single-cell level | <https://www.nature.com/articles/s41586-020-2157-4> |
| study41 | Homo sapiens | Esophagus - Construction of a human cell landscape at single-cell level | <https://www.nature.com/articles/s41586-020-2157-4> |
| study42 | Homo sapiens | Fallopian tube - Construction of a human cell landscape at single-cell level | <https://www.nature.com/articles/s41586-020-2157-4> |
| study43 | Homo sapiens | Gallbladder - Construction of a human cell landscape at single-cell level | <https://www.nature.com/articles/s41586-020-2157-4> |
| study44 | Homo sapiens | Heart - Construction of a human cell landscape at single-cell level | <https://www.nature.com/articles/s41586-020-2157-4> |
| study45 | Homo sapiens | Small intestine ileum - Construction of a human cell landscape at single-cell level | <https://www.nature.com/articles/s41586-020-2157-4> |
| study46 | Homo sapiens | Small intestine jejunum - Construction of a human cell landscape at single-cell level | <https://www.nature.com/articles/s41586-020-2157-4> |
| study47 | Homo sapiens | Kidney - Construction of a human cell landscape at single-cell level | <https://www.nature.com/articles/s41586-020-2157-4> |
| study48 | Homo sapiens | Liver - Construction of a human cell landscape at single-cell level | <https://www.nature.com/articles/s41586-020-2157-4> |
| study49 | Homo sapiens | Lung - Construction of a human cell landscape at single-cell level | <https://www.nature.com/articles/s41586-020-2157-4> |
| study50 | Homo sapiens | Muscle - Construction of a human cell landscape at single-cell level | <https://www.nature.com/articles/s41586-020-2157-4> |
| study51 | Homo sapiens | Omental adipose tissue - Construction of a human cell landscape at single-cell level | <https://www.nature.com/articles/s41586-020-2157-4> |
| study52 | Homo sapiens | Pancreas - Construction of a human cell landscape at single-cell level | <https://www.nature.com/articles/s41586-020-2157-4> |
| study53 | Homo sapiens | Peripheral blood - Construction of a human cell landscape at single-cell level | <https://www.nature.com/articles/s41586-020-2157-4> |
| study54 | Homo sapiens | Lung pleura - Construction of a human cell landscape at single-cell level | <https://www.nature.com/articles/s41586-020-2157-4> |
| study55 | Homo sapiens | Prostate - Construction of a human cell landscape at single-cell level | <https://www.nature.com/articles/s41586-020-2157-4> |
| study56 | Homo sapiens | Rectum - Construction of a human cell landscape at single-cell level | <https://www.nature.com/articles/s41586-020-2157-4> |
| study57 | Homo sapiens | Sigmoid colon - Construction of a human cell landscape at single-cell level | <https://www.nature.com/articles/s41586-020-2157-4> |
| study58 | Homo sapiens | Spleen - Construction of a human cell landscape at single-cell level | <https://www.nature.com/articles/s41586-020-2157-4> |
| study59 | Homo sapiens | Stomach - Construction of a human cell landscape at single-cell level | <https://www.nature.com/articles/s41586-020-2157-4> |
| study60 | Homo sapiens | Brain temporal lobe - Construction of a human cell landscape at single-cell level | <https://www.nature.com/articles/s41586-020-2157-4> |
| study61 | Homo sapiens | Thyroid - Construction of a human cell landscape at single-cell level | <https://www.nature.com/articles/s41586-020-2157-4> |
| study62 | Homo sapiens | Trachea - Construction of a human cell landscape at single-cell level | <https://www.nature.com/articles/s41586-020-2157-4> |
| study63 | Homo sapiens | Transverse colon - Construction of a human cell landscape at single-cell level | <https://www.nature.com/articles/s41586-020-2157-4> |
| study64 | Homo sapiens | Ureter - Construction of a human cell landscape at single-cell level | <https://www.nature.com/articles/s41586-020-2157-4> |
| study65 | Homo sapiens | Uterus - Construction of a human cell landscape at single-cell level | <https://www.nature.com/articles/s41586-020-2157-4> |
| study66 | Homo sapiens | SARS-CoV-2 receptor ACE2 and TMPRSS2 are predominantly expressed in a transient secretory cell type in subsegmental bronchial branches | <https://doi.org/10.15252/embj.20105114> |
| study67 | Homo sapiens | SARS-CoV-2 receptor ACE2 and TMPRSS2 are predominantly expressed in a transient secretory cell type in subsegmental bronchial branches | <https://doi.org/10.15252/embj.20105114> |
|  |  |  |  |
